# Preclinical safety assessment of photoluminescent metal quantum clusters stabilized with autologous serum proteins for host specific theranostics

**DOI:** 10.7150/ntno.82978

**Published:** 2023-03-27

**Authors:** Kritika Sood, Pranjali Yadav, Manu Jamwal, Reena Das, Asifkhan Shanavas

**Affiliations:** 1 Inorganic and Organic Nanomedicine Lab, Chemical Biology Unit, Institute of Nano Science and Technology, Sector-81, Mohali, Punjab, 140306, India; 2 Department of Haematology, Post Graduate Institute of Medical Education and Research (PGIMER), Madhya Marg, Sector 12, Chandigarh, 160012, India

## Abstract

Host derived serum proteome stabilised red-emitting gold quantum clusters (or Au-QC-NanoSera or QCNS) of size range ~2 nm have been synthesised in a first reported study. The host serum was taken from bovine, murine and human origins to establish the proof of concept. *In-vitro* biocompatibility with normal murine L929 fibroblast cells and radiosensitisation ability against PLC/PRF/5 hepatoma cells was established. A concentration dependant radiosensitisation effect of QCNS at differential γ-radiation doses was observed with almost 90% killing of cancer cells at a radiation dose of 5Gy. Acute and subacute safety, and non-immunogenicity of autologously derived QCNS was established in in-bred C57BL/6 mice. The biodistribution analysis revealed that the QCNS were effectively cleared from the body over a course of 28 days and were found to pose no major threat to the proper functioning and morphology of the mice.

## Introduction

With the widespread exploration of nanotechnology in the healthcare industry, novel multifunctional nanomaterials have been developed for the simultaneous diagnostic and therapeutic applications. One such class of nanomaterials is photoluminescent protein stabilised noble metal quantum clusters that have recently gained a lot of attention due to their ability to be used for simultaneous theranostic applications as well as their high biocompatibility. The protein stabilised metal quantum clusters are typically packed as core-shell type or metal-ligand type nanostructures wherein the protein shell acts as a scaffolding template as well as capping/stabilising agent for the metallic atoms such as Au, Ag, Cu, Pt. The bonding between the core metal atoms and the protein is usually through thiol containing amino acids like cysteine and is sterically protected due to the bulkiness of the protein 1. Protein stabilised gold nanoclusters (AuQC) depict smaller sizes (typically less than 3 nm) which in-turn confers them with a unique ability of photoluminescence due to quantum confinement. Due to their high biocompatibility, high photoluminescence, improved pharmacokinetics and fairly good *in vivo* clearance, AuQCs have been utilised in a wide variety of biological applications like cancer therapeutics including image guided therapeutics^2^, ^3^, drug delivery^4^, ^5^, and tumor targeting^6^, ^7^.

Radiation therapy is a very cost-effective cancer treatment as well as an efficient means of palliative care and is administered in ~50% of cancer patients 8, 9. Upon irradiation, the cancer cells are attenuated due to oxidative stress leading to cell membrane, protein and DNA damage. However, the healthy surrounding tissues and vital organs are also affected in the process. Hence, in order to enhance the selectivity of killing the cancer cells over the normal healthy cells, radiosensitizers such as AuQCs are being explored during radiation therapy^10^-^15^. The radiotherapy enhancement comes from the strong photoelectric absorption coefficient of gold nanoparticles wherein they transduce incoming high energy photons in to Compton electrons, photoelectrons, electron-positron pairs, and Auger electrons thereby leading to formation of free radicals such as ROS within cellular entities and leading to the killing of cancer cells 16. Apart from being effective radiosensitisers, atomically precise AuQCs have the advantage of high tumor uptake^13^, ^17^ and well established renal clearance^10^, ^18^, ^19^.

AuQCs have been previously fabricated using a wide range of proteins such as albumin^1^, ^20^, trypsin^21^, horseradish peroxidase^22^, chymotrypsin^23^, keratin, peptides 24-26, insulin^27^ as well as small biomolecules like cysteine^28^, ^29^ and glutathione^17^, ^30^. However, the reports for mixed protein stabilised AuQCs are limited. A mixture of lysozyme and albumin was utilized to enhance photoluminescence quantum yield of AuQCs due to Förster resonance energy transfer (FRET) between both the proteins 31. AuQC have also been synthesised using inexpensive naturally occurring proteinaceous sources such as chicken egg whites 32-34. Chicken egg whites (CEW) are complex mixed protein systems consisting of about 141 proteins including ovalbumin, ovotransferrin, ovomucoid, and lysozyme. CEW stabilized AuQCs have been utilised for developing an inexpensive H_2_O_2_ biosensor 32 as well as for selective and sensitive detection of Hg(II) ions 35. Another such complex multi-protein system is serum, which has not been explored for AuQC synthesis till now. Human serum, essentially without the clotting factors, possesses a rich pool of proteins hypothetically with potential to directly stabilize AuQCs. Serum typically consists of proteins - predominantly albumin (up to 70%), globulins and lipoproteins in addition to small biomolecules such as glucose and creatinine. Prior study utilized host derived serum protein to form nanovesicles for patient specific nanomedicines 36. Our group recently showed that drug nanocrystals can be directly stabilized with autologously derived serum proteins for cancer therapy 37. This approach takes advantage of the endogenously derived proteins that are less likely to cause inflammatory response in addition to being eventually metabolized safely by the body. Taking cues from this prior knowledge, we have fabricated AuQCs stabilized with whole serum proteome (Au-QC-NanoSera or QCNS) from bovine, murine and human origins. Acute toxicity, biodistribution and clearance of autologously derived QCNS in in-bred C57BL/6 mice were studied to establish their safety for potential *in vivo* applications. As a proof of concept, we have also demonstrated the capability of the QCNS to act as radiosensitizers.

## Experimental Section

### Materials

Gold (III) chloride trihydrate (520918; Sigma), fetal bovine serum (FBS, RM9955-heat inactivated; Himedia), bicinchoninic acid (BCA) protein assay kit (71285-3; Sigma), MTT (Thiazolyl blue tetrazolium bromide) dye for cell culture (TC191; Himedia), bovine serum albumin (BSA; Himedia), nuclear staining dye- bisBenzimide H33342 trihydrochloride (Hoechst 33342, B2261; Sigma), cytoskeletal staining dye- Phalloidin Tetramethylrhodamine B isothiocyanate (Phalloidin-TRITC, P1951; Sigma), standard lipopolysaccharide from E. coli 0111:B4 strain; TLR4 ligand (TLRL-EBLPS; InvivoGen). All other chemicals were of analytical grade and were used without further purification unless mentioned otherwise. Ultrapure water (~18.2 MΩ) was used for AuQC synthesis.

### Instrumentation

UV-Visible absorption spectra were recorded on Shimadzu UV-Vis spectrophotometer using 1 mL quartz cuvette. Fluorescence spectroscopy and TCSPC analysis was carried out using Fluorolog 3-221 fluorimeter equipped with 450 W Xenon lamp. Dynamic light scattering (DLS) and Zeta potential measurements were performed on Malvern Zetasizer Nano ZSP instrument. TEM measurements were performed using JEOL JEM-2100 at an accelerating voltage of 120 kV. Circular Dichroism was performed using J-15 model CD Spectrometer. XPS was carried out at CSIR-CECRI. Radiation exposure was carried out using Gamma radiator- Bhabhatron-II (Panacea Engineering Medicine) at INMAS, New Delhi. MALDI-TOF analysis was carried out at IIT-Bombay using Sinapinic acid (SA) and 2,5-Dihydroxybenzoic acid (DHB) matrix at 1:1 ratio.

### Synthesis of serum stabilized gold quantum cluster (Au-QC-NanoSera or QCNS)

**QCNS synthesis:** Quantum clusters stabilized with whole serum proteome from bovine, murine and human origins were synthesized as per previously reported method 1. Firstly, serum was quantified for total protein content using BCA Kit. Further, 5 mL of 50 mg/mL protein equivalent of serum was aliquoted in a clean glass vial and was allowed to pre-incubate at 37 °C for 15 mins. Aurochloric acid (10 mM, 5 mL) was added drop wise to the serum under high stirring (1000 rpm) and were allowed to incubate for 2-3 mins followed by adjusting the pH to ~11 using 1 M NaOH. The reaction was allowed to proceed for 24 hours in dark and sterile conditions. The QCNS thus formed were dialysed (MW cut off: 2000-14000 kDa) against ultrapure water for about 24 hrs and were stored at 4 °C for immediate analysis or lyophilized for long term storage. Post synthesis, bovine serum derived QCNS were used for all characterization including *in vitro* biocompatibility and radiosensitization studies. They were also used for evaluation of photoluminescence and size on a 1.5% agarose gel under UV-transilluminator using albumin stabilized AuQCs as a standard.

Human serum was used to showcase their ability to form H-QCNS. Healthy human serum was utilised after obtaining approval from the institutional ethics committee of PGIMER (PGI/IEC/2020/000787). For collection of human serum samples, 5ml blood was drawn from a healthy individual with prior informed consent. The blood was allowed to clot for 20-25 mins at room temperature and the samples were centrifuged at 5000 rpm for 10 mins at 4 °C. The serum was obtained as the supernatant and was stored at -20 °C in multiple aliquots until further use.

Murine serum was used to evaluate acute toxicity, biodistribution and clearance of autologously derived M-QCNS in in-bred C57BL/6 mice. Healthy mice serum was utilised after obtaining approval from the institutional ethics committee of IISER Mohali (IISERM/SAFE/PRT/2020/001). Collection and processing is explained in later section.

### Hemocompatibility studies

Hemocompatibility is one of the most critical parameters for design and safety evaluation of any nanomaterial, especially those injected intravenously. QCNS was challenged against red blood corpuscles derived from healthy human donor. Briefly, 5 mL blood was collected in a sterile vial pre-treated with trisodium citrate. 150 µL RBCs were incubated for 3 hours with 750 µL QCNS at different concentrations (12.5, 25, 50, 100 µg/ml in PBS) at 37 °C under mid shaking after which the RBCs were centrifuged [5000 rpm, 10mins at 4°C] to separate the lysed cellular component and the supernatant was evaluated for haemolytic activity. Milli-Q water and PBS were used as positive and negative controls for the assay. The experiment was performed in triplicates. The amount of hemoglobin was calculated by the following formula:

Amount of plasma Hb (mg/dL) 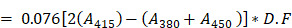


where A_380_, A_415_, and A_450_ are the absorbance values at 380, 415, and 450 nm, respectively. D.F is the dilution factor used for taking the absorbance. The percentage of hemolysis was calculated by the following equation:

% Hemolysis 
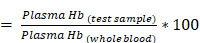


where plasma Hb _(test sample)_ and plasma Hb _(whole blood)_ correspond to the plasma hemoglobin value of test samples and the whole blood respectively.

The treated RBCs were fixed in 4% PFA solution and washed with PBS twice and dropcasted on a Si wafer for assessing their morphological analysis under SEM.

### Cellular studies

The cellular uptake and biocompatibility analysis of QCNS were carried out with murine fibroblast L929 cells. The radiosensitization studies were carried out using PLC/PRL/5 hepatoma cells at Institute of Nuclear Medicine and Advanced Science (INMAS), New Delhi. Both L929 and PLC/PRL/5 were cultured in DMEM supplemented with 10% FBS and 1% antibiotic antimycotic solution at 37 °C in a humidified incubator containing 5% CO_2_ with bi-weekly sub-culturing maintenance.

### *In-vitro* biocompatibility

For assessing the biocompatibility of QCNS, normal murine fibroblast L929 cells were seeded at a density of 5.0 x10^4^ cells in a 96 well plate for 24 hrs at 37 °C and 5% CO_2_. After reaching ~70% confluency, different concentrations of QCNS (0, 25, 50, 100 µg/ml) were incubated with the cells for 24hrs. Next day, the cells were washed with 1X PBS. Further, 10 µL of MTT solution (5mg/ml dissolved in PBS) diluted with 90 µL media was added and allowed to incubate for 4 hrs. Once the colour changed, the media was carefully removed and 100 µL of DMSO was added to each well and mixed properly until the purple formazan crystals were dissolved. The absorbance was recorded at 595 nm on a multimode plate reader. Cell viability percentage was calculated using the formula:


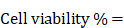


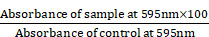


### Intracellular localisation study

To check the cellular uptake of QCNS, L929 cells were seeded on a cover slip at a density of 5x10^4^ cells per well in a 6 well plate for 24 hrs. Next day, QCNS was added at concentration of 300µg/ml and incubated for 24 hrs. The cells were further washed with 1x PBS thrice and fixed with 4% paraformaldehyde for about 15 mins. Next, the nuclei were counter stained with Hoechst 33342 for 10 min. After washing with PBS to remove excess stain, the cells were mounted on a glass slide and analyzed under LSM 880 Confocal microscope (Carl Zeiss AG, Germany).

### *In-vitro* radiosensitization studies

QCNS were investigated as a potent radiotherapy sensitizer on PLC/PRF/5 hepatoma cells. Briefly, 1x10^4^ cells were seeded and allowed to adhere overnight in a 96 well plate. The following day, the cells were treated with QCNS at 100 µg/ml and 50 µg/ml concentrations and incubated for 24 hrs. Next day, the cells were exposed to differential γ-radiation doses (0, 0.625, 1.25, 2.5 and 5 Gy) and further checked for cell viability using MTT assay. The experiment was performed in triplicate and the results were plotted as cell viability (percent of control).

### Assessment of γ-radiation induced reactive oxygen species

In order to assess the ROS generated due to QCNS catalysed radiosensitization, DCFDA assay was carried out. Dichlorodihydrofluorescein diacetate acetyl ester or H2DCFDA (a cell permeant non-fluorescent dye) is converted into its highly green fluorescent form 2',7'-dichlorofluorescein or DCF form after the removal of acetate groups by intracellular esterases and oxidation in the presence of ROS species. The green fluorescent signal is quantified *in-vitro* and is directly proportional to ROS species produced.

Briefly, PLC/PRF/5 cells were seeded at a density of 1x10^4^ cells/well in a 96 well cell culture plate and after allowing cells to adhere overnight they were treated with 100 µg/mL QCNS. The cells were irradiated with 5Gy γ-rays on the following day. After irradiation, the cells were washed with Hanks' Balanced Salt Solution and incubated with DCFDA (25 µg/ml) in serum free media for 4hrs. The fluorescence intensity of DCF (exc: 485 nm and em: 535 nm) was recorded at the start (0 min) and end point of the experiment (240 mins). The experiment was performed in triplicates and the results were plotted with respect to control.

### Assessing cellular damage post-radiation therapy

PLC/PRF/5 cells were seeded at a density of 5 × 10^4^ cells/well in a 6 well plate on cover slips. After adherence, the cells were incubated with 100 µg/mL QCNS for 24 hrs. This was followed by γ-ray irradiation of cells at a dose of 5 Gy. The cells were then washed in PBS twice to remove any traces of treatment and were fixed with 4% PFA for 10 mins at room temperature. Post-fixing, the cells were washed in PBS once to remove the excess PFA and the cellular nucleus was stained with Hoechst 33342 (2 μg/mL) for 15 min, followed by counterstaining the cytoskeleton with Phalloidin-TRITC for 20 min. The cells were washed twice with PBS to remove excess stain and mounted on to the cover slips. The nuclear and cytoskeletal damage were observed with a confocal laser scanning microscope (Carl Zeiss LSM 880, Germany) at 63x magnification.

### Animal experimentation

All the experimental procedures using laboratory animals were conducted with the approval of the Institutional Ethics Committee (IISERM/SAFE/PRT/2020/001) at IISER Mohali. For the biodistribution study, C57BL/6 female mice aged 6-8 weeks were divided in 7 groups with equal average weights (n=3): Control Day-1, Control Day-28, Untreated autologous serum Day-1 and Untreated autologous serum Day-28, M-QCNS Day-1 and M-QCNS Day-28 and Lipopolysaccharide Day-1. The mice were housed in cages that were located in a well-ventilated, temperature-controlled room with a light and dark period of 12 h each, with free access to water and food. Two weeks (i.e., **D_-14_**) prior to treatment, blood was collected from all the in-bred mice and serum was pooled (~2 mL) for the synthesis of M-QCNS. On day 0 **(D_0_)**, the mice were intravenously injected with single dose of M-QCNS (50mg/kg; Au 5mg/kg) or saline or untreated autologous serum or LPS (2mg/kg; positive control for acute inflammation). On day 14 **(D_14_)**, a second dose of saline, QCNS or untreated autologous serum was administered in the respective group. On day 1**(D_1_)** and day 28 **(D_28_)**, mice from each group were sacrificed for assessing acute inflammatory response and sub-acute toxicity respectively. Blood was collected and the serum was stored at -80°C for further biochemical analysis. Vital organs such as liver, spleen, kidneys, lungs and heart were extracted for biodistribution and histopathological analysis.

### Assessment of M-QCNS biodistribution

Vital organs including liver, spleen, lung, kidney, heart as well as excretory (faeces) samples were analysed for their Au content by treating them with ICP-MS grade nitric acid and incubated at 60 °C for 12 h. The solutions were then further diluted in deionized water such that an optimum pH 3-5 was obtained and were analysed using inductively coupled plasma mass spectrometer (Agilent 7900) to determine Au concentration against standard calibration curve obtained with solutions of ionic gold from 10 to 500 ppb.

### Acute inflammatory analysis

Acute inflammatory analysis was carried out for mice in following groups on day 1 post injection with M-QCNS, LPS and saline. After sacrificing the mice, the serum was separated from the clotted whole blood after 15mins and was analysed for 13 mouse cytokines including IL-1α, IL-1β, IL-6, IL-10, IL-12p70, IL-17A, IL-23, IL-27, MCP-1, IFN-β, IFN-γ, TNF-α, and GM-CSF using a fluorescent bead-based assay (LEGEND plex). These cytokines are majorly expressed by innate immune cells and are critical in understanding acute immune response to a foreign body such as M-QCNS in the systemic circulation.

### Biochemical analysis

The serum obtained from blood samples collected at D1 and D28 were analysed for biochemical parameters such as aspartate aminotransferase, alanine transaminase, creatinine and blood urea nitrogen using kits from ERBA Mannheim as per manufacturer's protocol.

### Histopathological analysis

Organs were carefully collected, fixed and conserved in 10% buffered formalin solution before paraffin-embedding. 5 µm thick paraffin sections of different organs were then processed manually and stained with haematoxylin and eosin dye. The slides were examined at different magnifications to assess any abnormal tissue microarchitecture under an inverted light microscope.

### Statistical Analysis

All graphs were plotted using OriginPro software. ANOVA and Student's t-test were performed for statistical significance analysis and the results were indicated as * p<0.05 and ** p<0.01 wherever required.

## Results and Discussion

### Characterisation of QCNS

**Figure 1:**
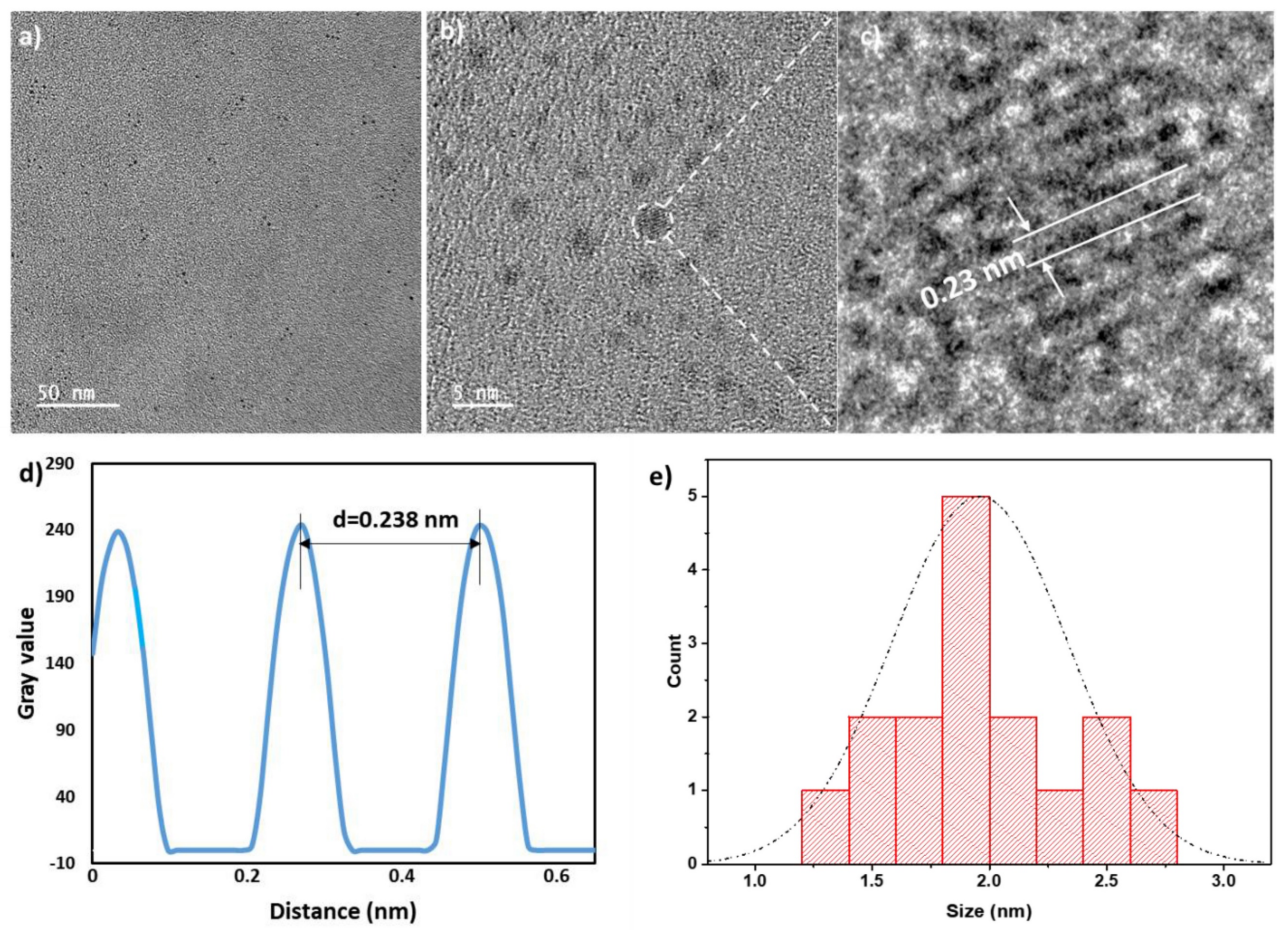
TEM micrograph of QCNS in a), b) depicting size distribution; and C) HR-TEM indicating typical Au d-spacing; d) Inverse FFT plot of QCNS and e) Size distribution of QCNSs using data from (a).

The size of dialyzed bovine derived QCNS (or simply QCNS) was observed to be about 1.9±0.3 nm with a polydispersity index of ~0.2 as characterized using transmission electron microscopy (Fig. 1). High resolution TEM confirmed the d-spacing of ~0.24 nm corresponding to the (111) crystal plane of the face centred cubic (fcc) lattice of metallic gold.

**Figure 2:**
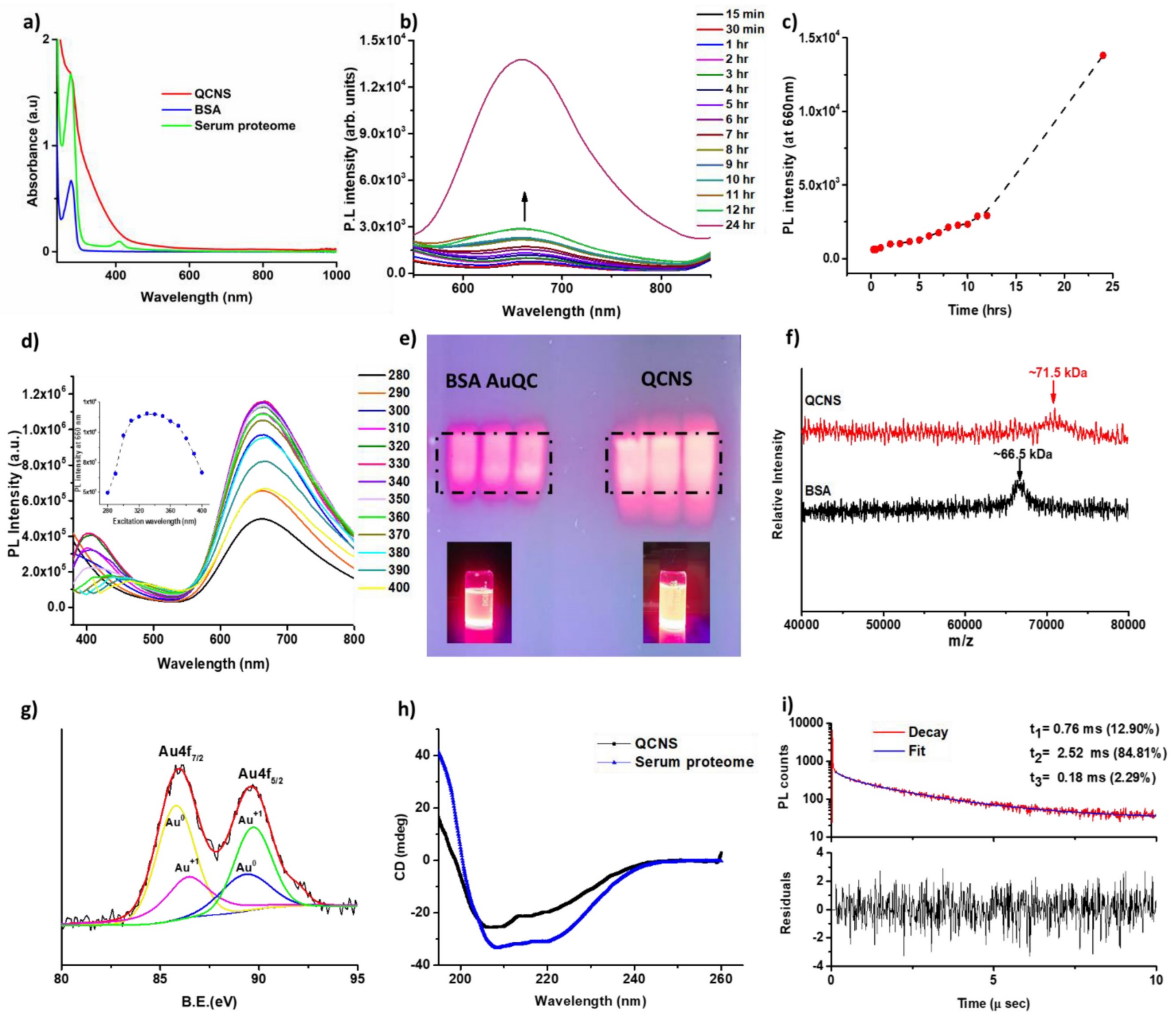
Optical characterisation of QCNS: a) UV-Vis absorbance peak in water; b) Time dependant photoluminescence spectra; c) PL intensity at λmax 660 nm as a function of time; d) Excitation dependant emission mapping of QCNS; e) Agarose gel electrophoresis of BSA-AuQCs; f) MALDI-TOF analysis; g) Deconvoluted XPS spectra; h) Circular dichroism analysis; and i) Time-correlated single photon counting (TCSPC) analysis of QCNS.

The absorption spectra of QCNS was devoid of any plasmon band typical observed in larger gold nanoparticles confirming presence of quasi molecular atomic clusters stabilized with serum proteins. A typical peak at ~280 nm denoted presence of aromatic amino acid residues-mainly tryptophan and tyrosine (Fig. 2a). The peak in serum proteome at ~410 nm is due to haemoglobin that might be present in trace levels in serum samples 38. There was a time dependant evolution of fluorescence from QCNS with an emission λ_max_ at ~ 660 nm after exciting at 340 nm (Fig. 2c) with a quantum yield of ~12.3% as measured with quinine sulphate as reference dye. There was no shift in the excitation dependent emission maxima for QCNS (Fig. 2d) and the maximum emission intensity was recorded at 330-340 nm. The fluorescence spectra of BSA and serum proteome are also depicted (Fig. S1a). Due to the heterogeneity of the QCNS, agarose gel electrophoresis was carried out where the band pattern was comparable to homogenous albumin stabilized AuQCs (Fig. 2e). Albumin is the major protein in serum and could possibly dominate reduction of entrapped gold ions to form QCNS at pH~12, over other proteins. The zeta potential for QCNS, serum proteome and BSA were all found to be negative indicating similar overall charge (Fig. S1b).

**Figure 3:**
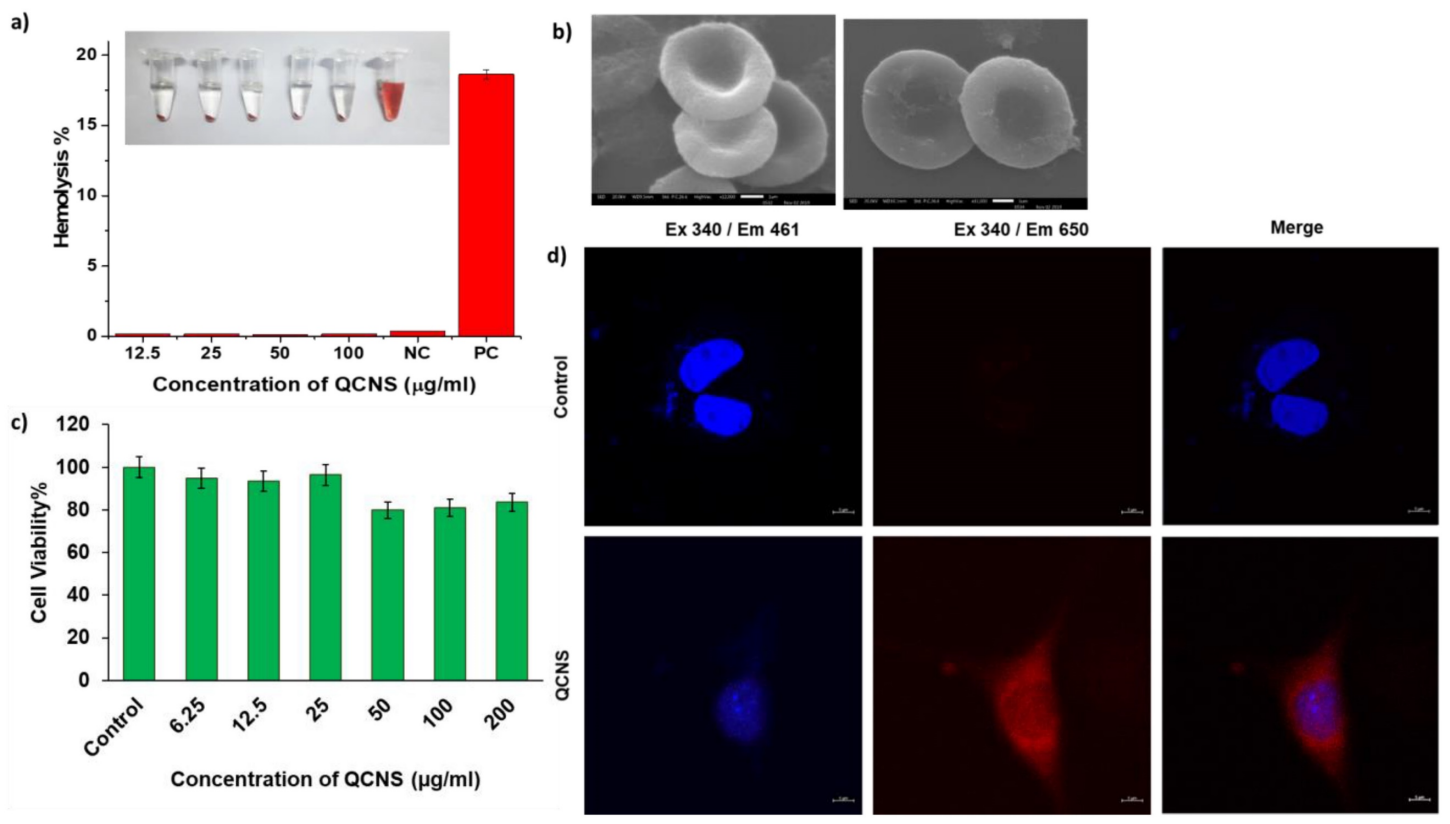
a) Hemocompatibility at different QCNS concentration [NC:Negative control; PC: Positive control]; Inset: Photograph showing hemolysis after incubation with different QCNS concentrations b) RBCs after interaction with QCNSs at 12.5 µg/ml and 100 µg/ml respectively c) Biocompatibility at different QCNS concentrations with normal L929 cells d) Red emissive QCNS uptaken by L929 cells as observed with confocal microscopy (magnification-63x).

The formation of QCNS stabilized predominantly by albumin was further confirmed with MALDI-TOF analysis (Fig. 3f). QCNS depicted a molecular weight of ~71.5 kDa, which is 5 kDa more than untreated albumin (~66.5 kDa). This difference in the molecular weight corresponds to ~25 atoms of gold present within QCNS, which is similar to the size of BSA-AuQCs 1*.* Furthermore, the oxidation state of gold within QCNS was assessed with X-ray photoelectron spectroscopy (Fig. 3g). The Au4f spectra was deconvoluted to reveal Au4f_7/2_ peaks centred at 85.81 eV and 86.5 eV corresponding to Au^0^ and Au^+1^ which are higher than reported for BSA- AuQCs 1. This might be attributed to the fact that QCNS contain several smaller clusters 39. This confirms that QCNS possess the classical core & shell architecture of Au & Au-thiol which is typical of AuQCs. The CD spectra of untreated serum proteome indicated an overall alpha helical conformation (Fig. 2h). However, with the formation of QCNS, the peaks at ~209 nm descended and peak at ~222 nm ascended, signifying the partial unfolding of conformation due to the newly emerged protein-gold bond and the associated structural rearrangements. These CD peaks at ~209 nm and ~222 nm corresponding to π → π* and n → π* electronic transition respectively are characteristic peaks of BSA 40.

Photoluminescence lifetime of a fluorophore is a measure of the time that it remains in excited state (S_1_ or T_1_) before emitting the energy in the form of a photon and relaxing back to its ground state (S_0_). The fluorescence decay curves for QCNS at ~340 nm revealed the average lifetime to be ~2.24 µs by integrating three components of ζ_1_ =0.76 µs (12.9%), ζ_2_ =2.52 µs (84.81%) and ζ_3_ =0.18 µs (2.29%) (Fig. 2i). The TCSPC analysis was carried out after dispersing QCNS in ultrapure water. Since the lifetimes were observed to be on the microsecond scale, the emission was predominantly due to phosphorescence^39^. Further, the basic optical properties of both Human derived QCNS (H-QCNS) and Murine derived QCNS (M-QCNS) were also found to be comparable (Fig. S2 and Fig. S3).

### Cellular studies

The QCNS were tested up to a concentration of 100 µg/ml against RBCs and found to be highly hemocompatible with <0.3% lysis as opposed to the haemolytic positive control (>5%) (Fig. 3a). The RBCs were also observed to have the QCNS adsorbed onto their surface with no obvious changes in the cellular structure (Fig. 3b). The toxicity assessment of QCNS against normal fibroblast L929 cells up to a concentration of 200 µg/ml revealed >80% cell viability, indicating their biocompatible nature (Fig. 3c). The cellular uptake of QCNS could be traced through their red emission within the cytoplasm (Fig. 3d).

**Figure 4:**
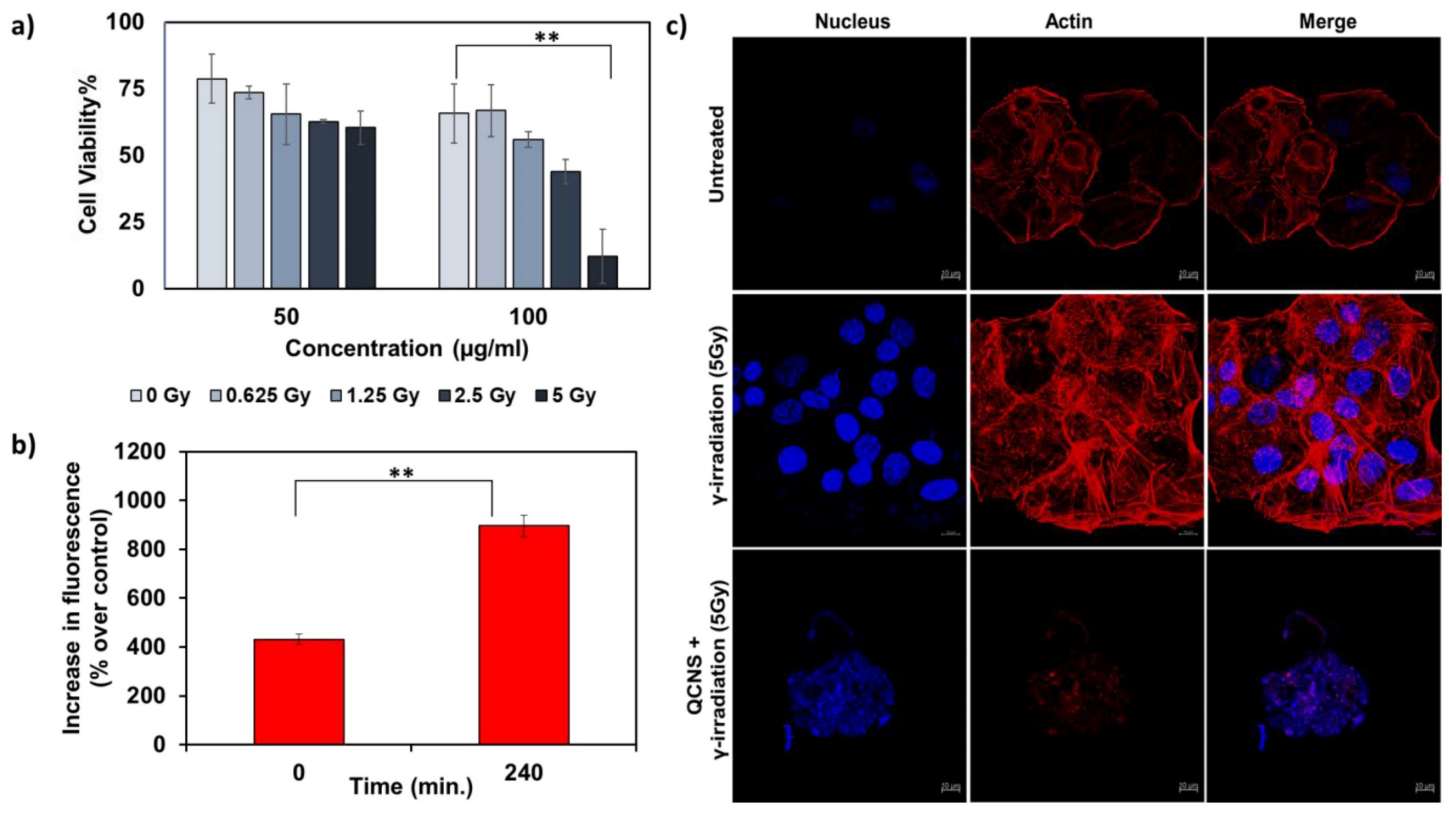
*In-vitro* radiation therapy a) Cytotoxicity observed at different radiation doses with 50 and 100µg/ml QCNS and b) Increase in ROS percentage post radiation treatment c) Confocal images of PP5 hepatoma cells treated with 5Gy radiation and QCNS+5Gy radiation. ** p <0.01.

We further investigated QCNS as radiosensitizers against PLC/PRF/5 hepatoma cells. Fig. 4a shows a concentration dependant radiosensitisation effect of QCNS at differential γ-radiation doses. At the highest radiation dose of 5Gy for 5mins, less than 10 % cancer cells survived. The IC50 value was found to be ~ 58µg/ml at 5Gy radiation dose. Further, DCF-DA assay indicated increase in the formation of reactive oxygen species up to 2 folds post radiosensitization with QCNS, in comparison to control (Fig. 4b). To visualise the effect caused by QCNS in conjunction with 5Gy radiation on the cellular morphology, we carried out confocal microscopic imaging of treated hepatoma cells stained with Hoechst-33342 and phalloidin-TRITC to analyse DNA and cytoskeleton respectively. As observed in Fig. 4c, the radiosensitized hepatoma cells showed complete destruction of cellular morphology with nuclear shrinkage and condensation in addition to disintegration of actin filaments leading to complete loss of cellular integrity. However, hepatoma cells treated with radiation alone showed minimal nuclear damage with no prominent cytoskeletal damage. These results signify high biocompatibility and significantly enhanced radiosensitization of the QCNS.

### Pre-clinical safety assessment and biodistribution of MQCNS

**Figure 5:**
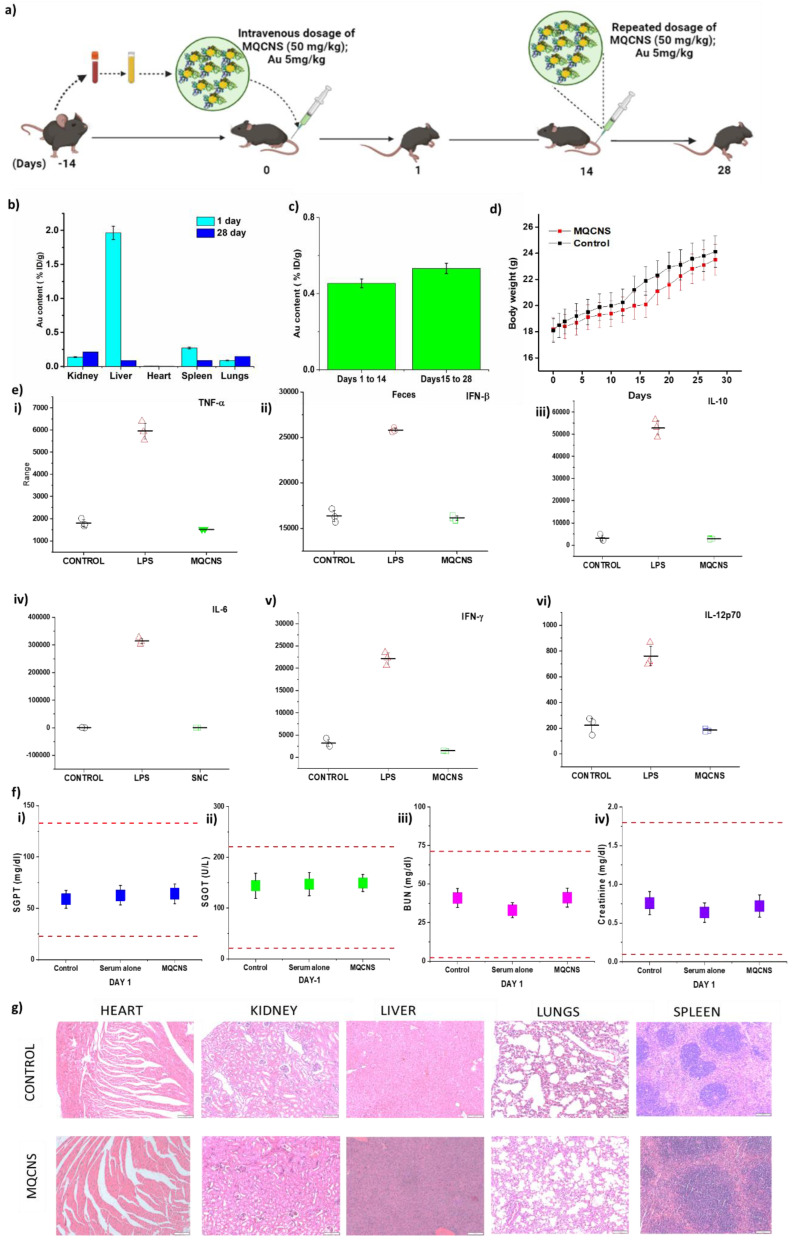
Pre-clinical safety and biodistribution analysis of MQCNS. Panels showing a) Schematic for *in-vivo* dosing for pre-clinical safety and biodistribution analysis; b) - c) Au biodistribution in vital organs and faeces; d) change in body weight; e) Acute inflammatory cytokine analysis for important cytokines (pg/ml above baseline). f) Vital organ functioning tests: SGPT, SGOT, BUN, Creatinine on Day 1 p.i. and g) Histopathological analysis on Day 1 post injection.

For all animal experimentation, murine derived QCNS (M-QCNS) were prepared as indicated in the methods section. The schedule of treatment is depicted in Fig. 5a. The Biodistribution analysis of M-QCNS post 24 hours of intravenous injection showed predominant accumulation in the liver followed by spleen, kidney and lungs. However on Day 28, all the organs including liver possessed only marginal quantity (0.08% ID/g for liver and spleen) of MQCNS. While prior reports show that kidney is the most preferred route of excretion for nanoparticles with size less than 6-8nm 41-43, albumin stabilized AuQCs is an exception due to its retention from passing through healthy glomerular basal membrane 44. While there is no single pathway of albumin degradation, most studies have reported that GI tract is a significant site of albumin catabolism (~ 40-70%) in normal individuals 45. Kidneys also play a minor role contributing < 15% *in vivo* albumin excretion in healthy individuals even in the absence of a renal disease. Renal catabolism of albumin consists of glomerular filtration and tubular reabsorption. Possible ways of further catabolism of this protein are lysosomal proteolysis to amino acids and short peptides, recycling of degradation products into the bloodstream and tubular lumen or transcytosis of whole molecules^46^. Involvement of liver is also reported contributing <10-15% of albumin catabolism in the body. Hence, no organ can be held majorly responsible for albumin catabolism. Prior research suggests the possibility of the albumin catabolism to be diffused process which can occur through RES (reticulo endothelial system). These immune cells have the ability to catabolize various proteins and are widely present as residential macrophages in all organs 47. Damaged protein including albumin are rapidly cleared from the serum via this pathway and blockade of the RES significantly slows the elimination of such altered albumin molecules. Presence of Au in faeces revealed that QCNS were excreted out from body cumulatively estimating to 0.43 % ID/g over a period of first two weeks (Day 1-14) and after second dosage, 0.53% ID/g Au cumulatively over the period of the following two weeks (Day 15-28).

Body weight of saline and M-QCNS injected mice showed no significant changes and was found to show increase with time depicting normal growth of mice (Fig. 5d). Acute inflammatory cytokine markers (Table S1) that are expressed due to immune responses were assessed on day-1. The results revealed no significant changes in the major inflammatory cytokine levels including TNF-α, IFN-β, IFN-ɤ, IL-6, IL-10, IL-12(p70) in comparison to positive control (LPS) group as shown in (Fig. 5e & Fig. S4). The serum biomarkers for proper functioning of vital organs were also analysed.

Aspartate aminotransferase (Reference range: 46-221 U/L), alanine aminotransferase (Reference range: 22-133 U/L), blood urea creatinine (Reference range: 2-71 mg/dl) and creatinine (Reference range: 0.1-1.8 mg/dl) were analysed post Day 1 and Day 28 of treatment. The test results were found to be within the normal reference range for C57BL/6 mice and therefore did not affect the vital organ functioning (Fig. 5f & Fig. S5A). Histopathological analysis of the organ with hematoxylin and eosin staining depicted no signs of infiltrating inflammatory cells, or tissue damage post 24hours (Fig. 5g) and 28 days (Fig. S5 B). Hence, the dual dosage regimen of autologously derived QCNS (50mg/kg; Au 5mg/kg per dose) was observed to be safe for further preclinical and clinical applications.

## Conclusion and Future Prospects

Host specific serum protein stabilised gold quantum clusters with ultra-small size, good aqueous solubility and photostability have been observed to act as radiosensitisers *in-vitro*. Preliminary examination indicated that albumin is predominantly involved in the formation of quantum clusters. However, the other major serum proteins such as globulins and lipoproteins also possess the basic chemical composition required to stabilize AuQCs. Future directions may include identification of the right synthetic environment to activate these proteins for QC stabilization and eventual utilization for targeted theranostics. In general, the QCNS seem to be safe after double dosage intravenous administration indicating their potential for clinical translation. Considering their host specific nature, QCNS could be utilized in a heterogenous population that fail to tolerate generic medicinal approaches. Further evaluation of QCNS for long term biodistribution and simultaneous validation of their excretory pathway would pave way for their application in personalized theranostics.

## Supplementary Material

Supplementary figures and table.

## References

[1] Xie J, Zheng Y, Y (2009). Ying J. Protein-Directed Synthesis of Highly Fluorescent Gold Nanoclusters. J. Am. Chem. Soc.

[2] Hu D-H, Sheng Z-H, Zhang P-F, Yang D-Z, Liu S-H, Gong P, Gao D-Y, Fang S-T, Ma Y-F, Cai L-T (2013). Hybrid gold-gadolinium nanoclusters for tumor-targeted NIRF/CT/MRI triple-modal imaging *in vivo*. Nanoscale.

[3] Sood K, Shanavas A (2021). The Role of Gold Nanoclusters as Emerging Theranostic Agents for Cancer Management. Curr. Pathobiol. Rep.

[4] Khandelia R, Bhandari S, Pan UN, Ghosh SS, Chattopadhyay A (2015). Gold Nanocluster Embedded Albumin Nanoparticles for Two-Photon Imaging of Cancer Cells Accompanying Drug Delivery. Small.

[5] Chen D, Luo Z, Li N, Lee JY, Xie J, Lu J (2013). Amphiphilic Polymeric Nanocarriers with Luminescent Gold Nanoclusters for Concurrent Bioimaging and Controlled Drug Release. Adv. Funct. Mater.

[6] Tang S, Peng C, Xu J, Du B, Wang Q, Vinluan 3rd RD, Yu M, Kim MJ, Zheng J (2016). Tailoring Renal Clearance and Tumor Targeting of Ultrasmall Metal Nanoparticles with Particle Density. Angew. Chem. Int. Ed. Engl.

[7] Zhang P, Yang XX, Wang Y, Zhao NW, Xiong ZH, Huang CZ (2014). Rapid synthesis of highly luminescent and stable Au20 nanoclusters for active tumor-targeted imaging *in vitro* and *in vivo*. Nanoscale.

[8] Atun R, Jaffray DA, Barton MB, Bray F, Baumann M, Vikram B, Hanna TP, Knaul FM, Lievens Y, Lui TYM, Milosevic M, O'Sullivan B, Rodin DL, Rosenblatt E, Van Dyk J, Yap ML, Zubizarreta E, Gospodarowicz M (2015). Expanding global access to radiotherapy. Lancet Oncol.

[9] Abdel-Wahab M, Gondhowiardjo SS, Rosa AA, Lievens Y, El-Haj N, Polo Rubio JA, Prajogi G Ben, Helgadottir H, Zubizarreta E, Meghzifene A, Ashraf V, Hahn S, Williams T, Gospodarowicz M (2021). Global Radiotherapy: Current Status and Future Directions—White Paper. JCO Glob. Oncol.

[10] Luo D, Wang X, Zeng S, Ramamurthy G, Burda C, Basilion JP (2019). Targeted Gold Nanocluster-Enhanced Radiotherapy of Prostate Cancer. Small.

[11] Ghahremani F, Kefayat A, Shahbazi-Gahrouei D, Motaghi H, Mehrgardi MA, Haghjooy-Javanmard S (2018). AS1411 aptamer-targeted gold nanoclusters effect on the enhancement of radiation therapy efficacy in breast tumor-bearing mice. Nanomedicine.

[12] Liang G, Jin X, Zhang S, Xing D (2017). RGD peptide-modified fluorescent gold nanoclusters as highly efficient tumor-targeted radiotherapy sensitizers. Biomaterials.

[13] Zhang X-D, Luo Z, Chen J, Shen X, Song S, Sun Y, Fan S, Fan F, Leong DT, Xie J (2014). Ultrasmall Au10-12(SG)10-12 Nanomolecules for High Tumor Specificity and Cancer Radiotherapy. Adv. Mater.

[14] Samani RK, Tavakoli MB, Maghsoudinia F, Motaghi H, Hejazi SH, Mehrgardi MA (2020). Trastuzumab and folic acid functionalized gold nanoclusters as a dual-targeted radiosensitizer for megavoltage radiation therapy of human breast cancer. Eur. J. Pharm. Sci.

[15] Jia T-T, Yang G, Mo S-J, Wang Z-Y, Li B-J, Ma W, Guo Y-X, Chen X, Zhao X, Liu J-Q, Zang S-Q (2019). Atomically Precise Gold-Levonorgestrel Nanocluster as a Radiosensitizer for Enhanced Cancer Therapy. ACS Nano.

[16] Butterworth KT, McMahon SJ, Currell FJ, Prise KM (2012). Physical basis and biological mechanisms of gold nanoparticle radiosensitization. Nanoscale.

[17] Zhang X-D, Luo Z, Chen J, Song S, Yuan X, Shen X, Wang H, Sun Y, Gao K, Zhang L, Fan S, Leong DT, Guo M, Xie J (2015). Ultrasmall Glutathione-Protected Gold Nanoclusters as Next Generation Radiotherapy Sensitizers with High Tumor Uptake and High Renal Clearance. Sci. Rep.

[18] Zhang X-D, Chen J, Luo Z, Wu D, Shen X, Song S-S, Sun Y-M, Liu P-X, Zhao J, Huo S, Fan S, Fan F, Liang X-J, Xie J (2014). Enhanced Tumor Accumulation of Sub-2 nm Gold Nanoclusters for Cancer Radiation Therapy. Adv. Healthc. Mater.

[19] Liang G, Ye D, Zhang X, Dong F, Chen H, Zhang S, Li J, Shen X, Kong J (2013). One-pot synthesis of Gd3+-functionalized gold nanoclusters for dual model (fluorescence/magnetic resonance) imaging. J. Mater. Chem. B.

[20] Rajamanikandan R, Ilanchelian M (2019). Red emitting human serum albumin templated copper nanoclusters as effective candidates for highly specific biosensing of bilirubin. Mater. Sci. Eng. C.

[21] Liu J-M, Chen J-T, Yan X-P (2013). Near Infrared Fluorescent Trypsin Stabilized Gold Nanoclusters as Surface Plasmon Enhanced Energy Transfer Biosensor and *in vivo* Cancer Imaging Bioprobe. Anal. Chem.

[22] Wen F, Dong Y, Feng L, Wang S, Zhang S, Zhang X (2011). Horseradish Peroxidase Functionalized Fluorescent Gold Nanoclusters for Hydrogen Peroxide Sensing. Anal. Chem.

[23] Liu P, Shang L, Li H, Cui Y, Qin Y, Wu Y, Hiltunen JK, Chen Z, Shen J (2014). Synthesis of fluorescent α-chymotrypsin A-functionalized gold nanoclusters and their application to blot-based technology for Hg2+ detection. RSC Adv.

[24] Li Q, Pan Y, Chen T, Du Y, Ge H, Zhang B, Xie J, Yu H, Zhu M (2018). Design and mechanistic study of a novel gold nanocluster-based drug delivery system. Nanoscale.

[25] Vankayala R, Kuo C-L, Nuthalapati K, Chiang C-S, Hwang KC (2015). Nucleus-Targeting Gold Nanoclusters for Simultaneous *In vivo* Fluorescence Imaging, Gene Delivery, and NIR-Light Activated Photodynamic Therapy. Adv. Funct. Mater.

[26] Li Y, Yuan M, Khan AJ, Wang L, Zhang F (2019). Peptide-gold nanocluster synthesis and intracellular Hg2+ sensing. Colloids Surfaces A Physicochem. Eng. Asp.

[27] Liu C-L, Wu H-T, Hsiao Y-H, Lai C-W, Shih C-W, Peng Y-K, Tang K-C, Chang H-W, Chien Y-C, Hsiao J-K, Cheng J-T, Chou P-T (2011). Insulin-Directed Synthesis of Fluorescent Gold Nanoclusters: Preservation of Insulin Bioactivity and Versatility in Cell Imaging. Angew. Chemie Int. Ed.

[28] Deng H-H, Wu G-W, He D, Peng H-P, Liu A-L, Xia X-H, Chen W (2015). Fenton reaction-mediated fluorescence quenching of N-acetyl-l-cysteine-protected gold nanoclusters: analytical applications of hydrogen peroxide, glucose, and catalase detection. Analyst.

[29] Gao G, Chen R, He M, Li J, Li J, Wang L, Sun T (2019). Gold nanoclusters for Parkinson's disease treatment. Biomaterials.

[30] Wang J-Y, Chen J, Yang J, Wang H, Shen X, Sun Y-M, Guo M, Zhang X-D (2016). Effects of surface charges of gold nanoclusters on long-term *in vivo* biodistribution, toxicity, and cancer radiation therapy. Int. J. Nanomedicine.

[31] Mohanty JS, Baksi A, Lee H, Pradeep T (2015). Noble metal clusters protected with mixed proteins exhibit intense photoluminescence. RSC Adv.

[32] Li M, Yang D-P, Wang X, Lu J, Cui D (2013). Mixed protein-templated luminescent metal clusters (Au and Pt) for H2O2 sensing. Nanoscale Res. Lett.

[33] Tian J, Yan L, Sang A, Yuan H, Zheng B, Xiao D (2014). Microwave-Assisted Synthesis of Red-Light Emitting Au Nanoclusters with the Use of Egg White. J. Chem. Educ.

[34] Joseph D, Geckeler KE (2014). Synthesis of highly fluorescent gold nanoclusters using egg white proteins. Colloids Surfaces B Biointerfaces.

[35] Li X-J, Ling J, Han C-L, Chen L-Q, Cao Q-E, Ding Z-T (2017). Chicken Egg White-stabilized Au Nanoclusters for Selective and Sensitive Detection of Hg(II). Anal. Sci.

[36] Lazarovits J, Chen YY, Song F, Ngo W, Tavares AJ, Zhang Y-N, Audet J, Tang B, Lin Q, Tleugabulova MC, Wilhelm S, Krieger JR, Mallevaey T, Chan WCW (2019). Synthesis of Patient-Specific Nanomaterials. Nano Lett.

[37] Mimansa Jamwal M, Das R Shanavas A (2022). High Drug Loading Nanoparticles Stabilized with Autologous Serum Proteins Passively Inhibits Tumor Growth. Biomacromolecules.

[38] Farrell C-JL, Carter AC (2016). Serum indices: managing assay interference. Ann. Clin. Biochem.

[39] Tang Z, Chen F, Wang D, Xiong D, Yan S, Liu S, Tang H (2022). Fabrication of avidin-stabilized gold nanoclusters with dual emissions and their application in biosensing. J. Nanobiotechnology.

[40] Li D, Zhu M, Xu C, Ji B (2011). Characterization of the baicalein-bovine serum albumin complex without or with Cu2+or Fe3+ by spectroscopic approaches. Eur. J. Med. Chem.

[41] Longmire M, Choyke PL, Kobayashi H (2008). Clearance properties of nano-sized particles and molecules as imaging agents: considerations and caveats. Nanomedicine (Lond).

[42] Rengan AK, Bukhari AB, Pradhan A, Malhotra R, Banerjee R, Srivastava R, De A (2015). *In vivo* Analysis of Biodegradable Liposome Gold Nanoparticles as Efficient Agents for Photothermal Therapy of Cancer. Nano Lett.

[43] Yadav P, Mimansa, Munawara R, Kapoor K, Chaturvedi S, Kailasam K, Biswas SK, Bahadur D, Srivastava R, Mishra AK, Shanavas A (2022). Nontoxic *In vivo* Clearable Nanoparticle Clusters for Theranostic Applications. ACS Biomater. Sci. Eng.

[44] KOLTUN M, COMPER WD (2004). Retention of Albumin in the Circulation is Governed by Saturable Renal Cell-Mediated Processes. Microcirculation.

[45] WALDMANN TA (1977). ALBUMIN CATABOLISM. In: VM ROSENOER,M ORATZ,MABT-AS ROTHSCHILD Function and Uses, eds. Pergamon.

[46] Gburek J, Gołąb K, Juszczyńska K (2011). [Renal catabolism of albumin - current views and controversies]. Postepy Hig. Med. Dosw. (Online).

[47] KATZ J, ROSENFELD S, SELLERS AL (1961). Sites of plasma albumin catabolism in the rat. Am. J. Physiol.

