# Supplementary material for: Preclinical safety assessment of photoluminescent metal quantum clusters stabilized with autologous serum proteins for host specific theranostics
Source: Nanotheranostics. 2023 Mar 27;7(3):316–26. doi: 10.7150/ntno.82978 (PMC10093414; doi:10.7150/ntno.82978)
Supplement: Supplementary file 1 — Supplementary figures and table. [file ntnov07p0316s1.pdf]

Supplementary information:

Preclinical safety assessment of photoluminescent metal quantum clusters stabilized with autologous serum proteins for host specific theranostics

Kritika Sood[1], Pranjali Yadav[1], Manu Jamwal[2], Reena Das[2], Asifkhan Shanavas[1*]

[1]Inorganic and Organic Nanomedicine Lab, Chemical Biology Unit, Institute of Nano Science and Technology, Sector-81, Mohali, Punjab, 140306, India

[2]Department of Haematology, Post Graduate Institute of Medical Education and Research (PGIMER), Madhya Marg, Sector 12, Chandigarh, 160012, India

Zeta Potential and Photoluminescence spectra:

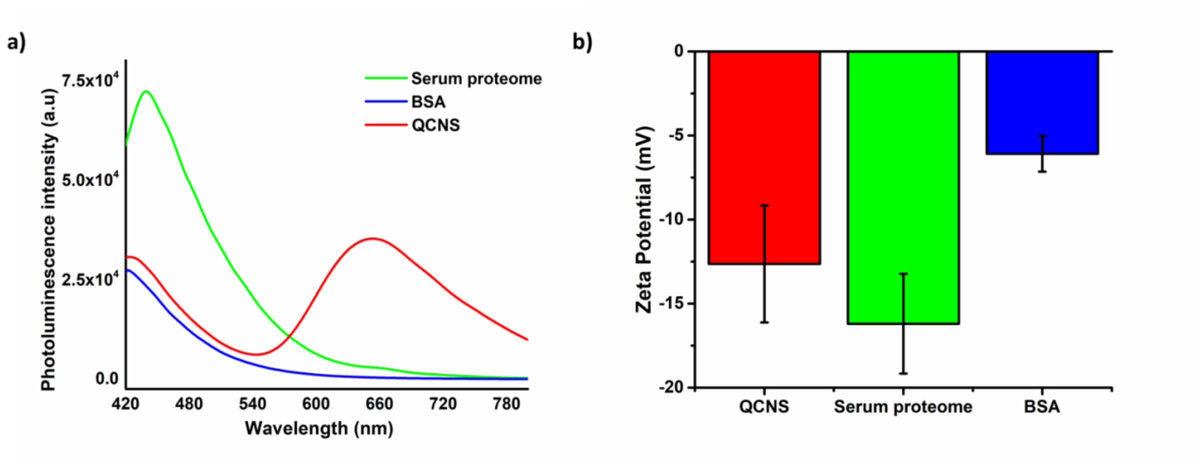

Fig S1: a) Photoluminescence spectra (λ_{ex}: 365 nm) and b) Zeta potential of QCNS (red), serum proteome (green) and BSA (blue) respectively.

Human QCNS (H-QCNS) characterisation and optical properties:

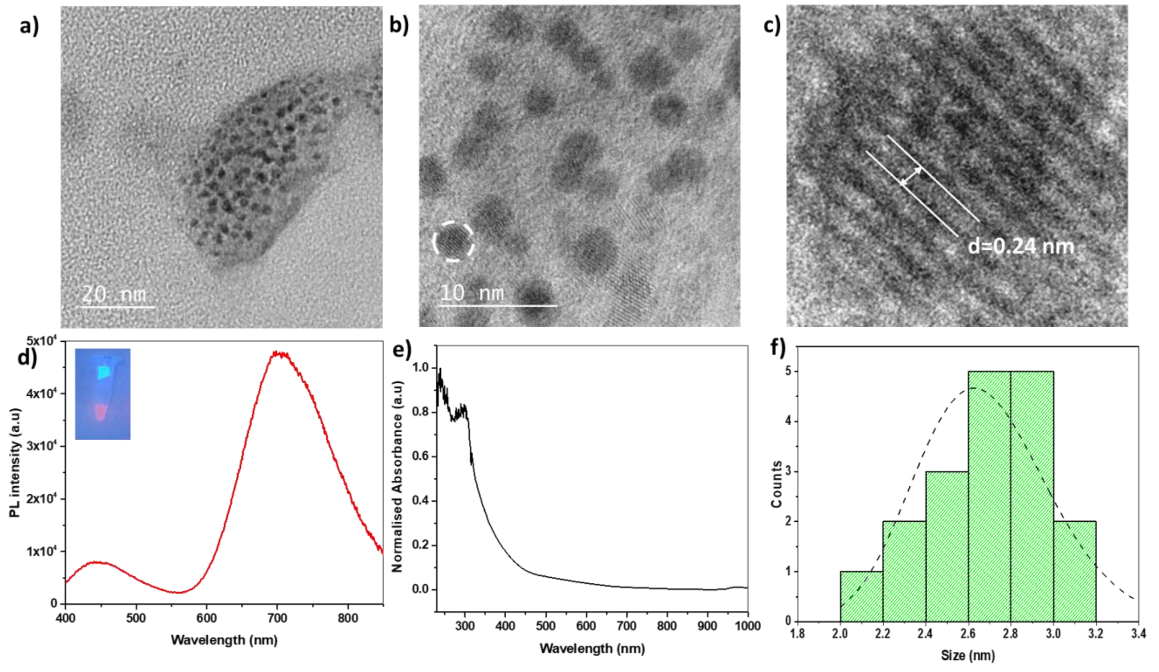

Fig S2: H-QCNS characterisation panel: a-b) TEM micrograph of as synthesised H-QCNS at different magnifications c) HR-TEM depicting d spacing=0.24 nm Emission spectra of H-QCNS at λex=365 nm (inset) Red fluorescent H-QCNS under UV-chamber d) UV-Vis absorbance f)Size distribution histogram obtained from TEM images depicting H-QCNS size ~2.68±0.3 nm

Murine QCNS (M-QCNS) characterisation and optical properties:

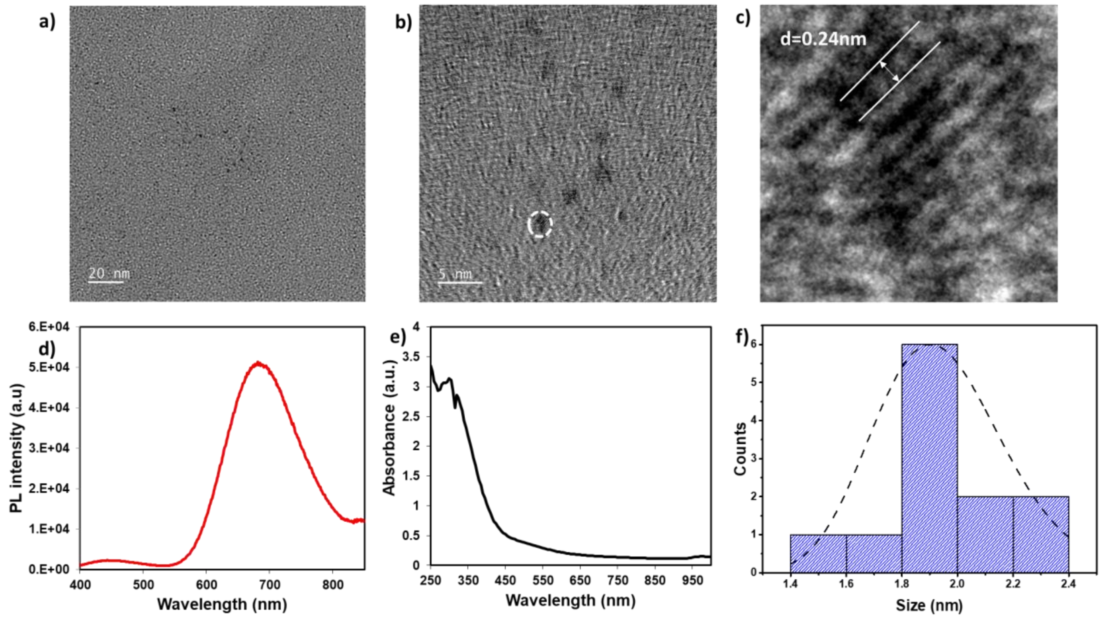

Fig S3: M-QCNS characterisation panel: a) and b) TEM micrograph of M-QCNS after negative staining with 1% uranyl acetate at different magnifications c) HR-TEM depicting d-spacing=0.24nm of Au for encircled QC in b) image. d) Emission spectra of as synthesised M-QCNS at λex=365 nm and e) UV-vis absorption spectra depicting no SPR of gold nanoparticles indicating formation of only gold nanoclusters. f) Size distribution histogram obtained from TEM images depicting M-QCNS size ~ 1.94±0.23 nm.

Table S1: Table depicting inflammatory cytokines analysed during the study and their role in inflammation.

Sr. No	Name of inflammatory Cytokine	Status	Immune response	Function
1.	IL-1α	Pro-inflammatory	innate	stimulates the activity of genes involved in inflammation and immunity
2.	IL-1β	Pro-inflammatory	innate	activated in pain, inflammation, autoimmune reaction
3.	IL-6	Both pro- and anti-inflammatory	Innate and adaptive	Pivotal cytokine in host immune response, induce acute phase response
4.	IL-10	anti-inflammatory	Innate and adaptive	inhibits the production of proinflammatory cytokines
5.	IL-12(p70)	Pro-inflammatory	adaptive	Promotes induction of TH_1 cells and cytotoxic T cell responses, enhances IFN- γ production
6.	IL-17A	Pro-inflammatory	Innate and adaptive	promoting recruitment of neutrophils to sites of inflammation
7.	IL-23	Pro-inflammatory	Innate and adaptive	Enhancing differentiation of TH1 cells and promoting inflammatory response in various organs
8.	IL-27	Both pro- and anti-inflammatory	Innate and adaptive	involved in T cell differentiation, inflammation and infection
9.	MCP-1	Chemokine	innate	directs the migration of monocytes and macrophages into inflammatory sites
10.	IFN-β	Both pro- and anti-inflammatory	innate	Induction and activation of transcription proteins to regulate inflammation
11.	IFN-γ	Pro-inflammatory	Innate and adaptive	triggers immune response activation and stimulation for pathogen clearance
12.	TNF-α	Pro-inflammatory	innate	Pivotal role in vasodilation and edema formation, signalling cascade leading to apoptosis/ necrosis
13.	GM-CSF	Pro-inflammatory	innate	Promotes growth and differentiation of granulocytes and macrophage cells

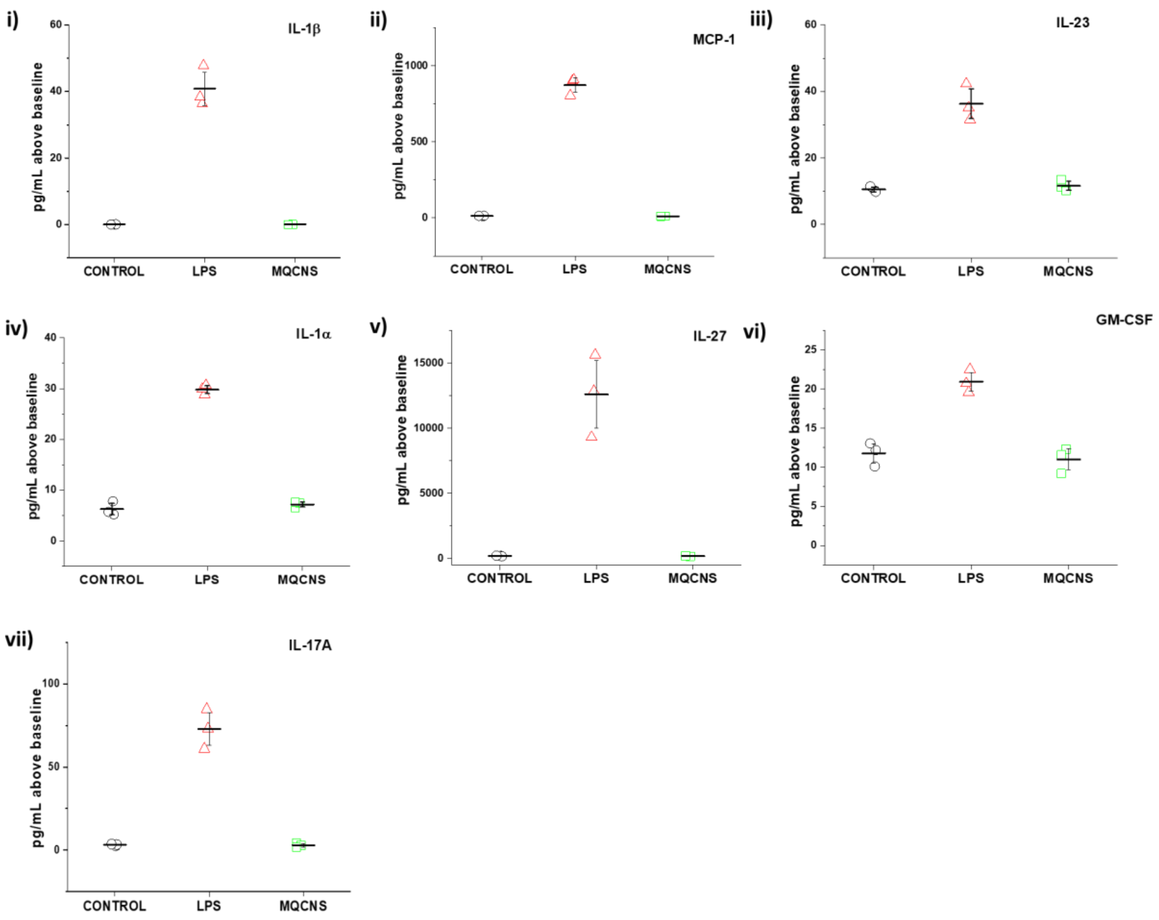

Fig S4: **Mouse Inflammatory cytokine analysis on Day 1 post injection:** (i-vii) IL-1β, MCP-1, IL-23, IL-1α, IL-27, GM-CSF and IL-17A. All values are given as pg/ml above baseline.

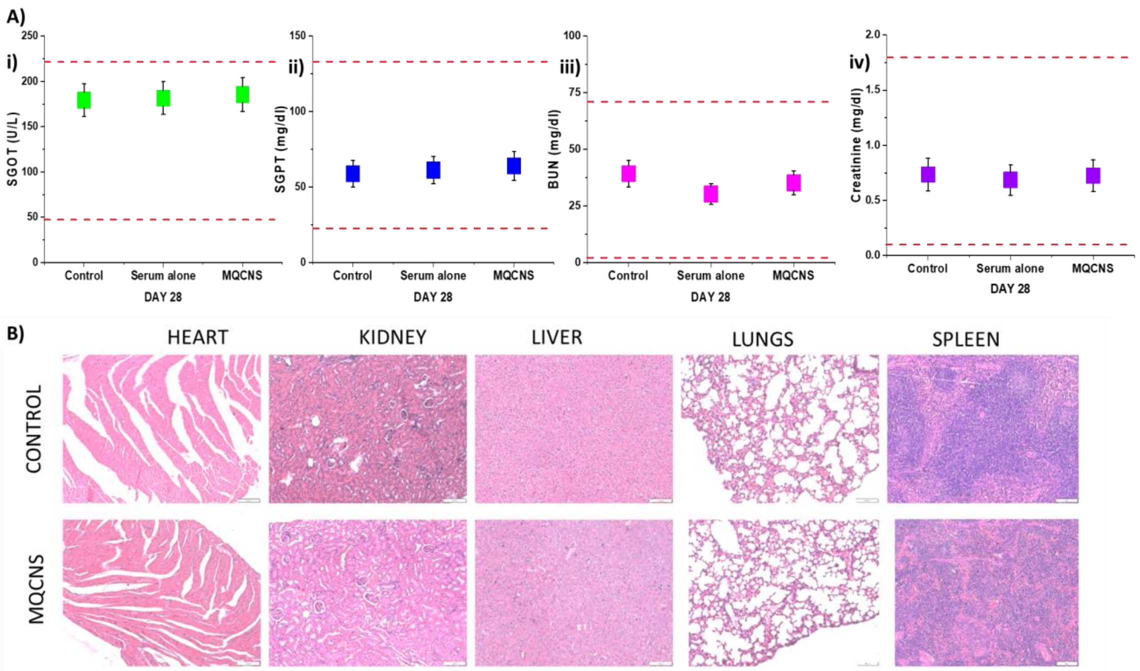

Fig S5: Preclinical safety analysis on 28th day post injection: A) Vital organ functioning tests: SGOT, SGPT, BUN, Creatinine and g) Histopathological analysis of vital organs on Day 28 post injection.
